# Hormone replacement therapy attenuates hearing loss: Mechanisms involving estrogen and the IGF‐1 pathway

**DOI:** 10.1111/acel.12939

**Published:** 2019-03-07

**Authors:** Tanika T. Williamson, Bo Ding, Xiaoxia Zhu, Robert D. Frisina

**Affiliations:** ^1^ Departments of Chemical & Biomedical and Medical Engineering, Global Center for Hearing & Speech Research University of South Florida Tampa Florida; ^2^ Departments of Communication Sciences & Disorders, Global Center for Hearing & Speech Research University of South Florida Tampa Florida

**Keywords:** age‐related hearing loss, aging, auditory system, hormone replacement therapy, HRT, neurodegeneration

## Abstract

Estradiol (E) is a multitasking hormone that plays a prominent role in the reproductive system, and also contributes to physiological and growth mechanisms throughout the body. Frisina and colleagues have previously demonstrated the beneficial effects of this hormone, with E‐treated subjects maintaining low auditory brainstem response (ABR) thresholds relative to control subjects (*Proceedings of the National Academy of Sciences of the United States of America*, 2006;103:14246; *Hearing Research,* 2009;252:29). In the present study, we evaluated the functionality of the peripheral and central auditory systems in female CBA/CaJ middle‐aged mice during and after long‐term hormone replacement therapy (HRT) via electrophysiological and molecular techniques. Surprisingly, there are very few investigations about the side effects of HRT in the auditory system after it has been discontinued. Our results show that the long‐term effects of HRT are permanent on ABR thresholds and ABR gap‐in‐noise (GIN) amplitude levels. E‐treated animals had lower thresholds and higher amplitude values compared to other hormone treatment subject groups. Interestingly, progesterone (P)‐treated animals had ABR thresholds that increased but amplitude levels that remained relatively the same throughout treatment. These results were consistent with qPCR experiments that displayed high levels of IGF‐1R in the stria vascularis (SV) of both E and P animal groups compared to combination treatment (E + P) animals. IGF‐1R plays a vital role in mediating anti‐apoptotic responses via the PI3K/AKT pathway. Overall, our findings gain insights into the neuro‐protective properties of E hormone treatments as well as expand the scientific knowledge base to help women decide whether HRT is the right choice for them.

## 1 INTRODUCTION

Hormone replacement therapy (HRT) is the most commonly sought out therapeutic intervention to relieve menopausal symptoms. Consequently, researchers and clinicians have been assessing the benefits and risks of HRT. Studies such as the Woman's Health Initiative (WHI) and Heart Estrogen‐Progestin Replacement Study (HERs) have evaluated the effects HRT has on blood pressure, breast cancer, cervical cancer, osteoporosis, stroke, and heart disease for menopausal women (Hulley et al., 1998; Ness, Aronow, Newkirk, & McDanel, 2005). The controversial findings from these studies swayed the decision of many women that were considering or currently undergoing HRT to avoid or discontinue treatment. Despite this, there are still millions of middle‐aged women in the United States and worldwide who are presently undergoing hormone therapy in an effort to relieve severe menopausal symptoms, aid in limiting osteoporosis, and reduce the risk of colon cancer (Ness et al., 2005). Most recently, research has indicated an additional benefit of certain types of hormone therapy—hearing protection. There have been various reports of estradiol (E) hormone therapy improving distortion product otoacoustic emissions (DPOAEs) as well as auditory brainstem response (ABR) amplitudes and latencies (Coleman, Campbell, Cooper, Welsh, & Moyer, 1994; Curhan et al., 2017; Milon et al., 2018; Price, Zhu, Guimaraes, Vasilyeva, & Frisina, 2009; Zhang et al., 2018). For example, Barati et al. (2013) reported that after 30 days of E hormone treatment patients with conductive hearing loss displayed improvements in ABR thresholds compared to control subjects. Contrarily, combination treatment (E + progesterone [P]) has been shown to have a negative effect on auditory function. Some studies have suggested that P is the negative component in the E + P combination, since combination treatment has detrimental effects on auditory processing. In 2006, Guimaraes et al. reported that P was the negative component for the E + P treatment duo since women who were treated with E + P had significantly higher ABR thresholds than E‐treated and age‐matched control patients. Their study concluded that the beneficial neural excitability activated by E may be counterbalanced by P's activation of inhibitory gamma‐aminobutyric acid (GABA) neurons. The specific actions of P on the auditory system, that is, benefits and/or risks of administering P alone, are still unknown.

Although most studies have focused on the influence of HRT during treatment, no studies have specifically evaluated hearing changes after hormone therapy has ended. Researchers have yet to fully understand the health benefits and risks of discontinuing hormone therapy, that is, are its effects reversible? In light of this, our study was designed to evaluate the hearing of ovariectomized (OVX) female CBA/CaJ middle‐aged mice undergoing HRT for a period of 6 months, and then after discontinuing treatment for 1 month. Subjects were randomly placed into hormone groups: E, P, E + P, and placebo (Pb). The full effects of P on hearing are still quite understudied as previously mentioned, which is why a unique P‐only group was incorporated here. ABR and ABR gap‐in‐noise (GIN) are electrophysiological techniques used to record and analyze the responses of auditory nerve and brainstem activity and temporal processing, respectively. To further expand on the results obtained for these electrophysiology hearing tests, molecular biology experiments were performed to evaluate the expression of insulin growth factor 1 receptor (IGF‐1R) and forkhead box O3 (FoxO3) in the cochlea during hormone therapy, from both in vitro and in vivo perspectives. Both of these gene pathways play significant roles in influencing the PI3K/AKT pathway and were specifically chosen because of their involvement in anti‐apoptotic responses and cell survival. The stria vascularis (SV) is a specialized cochlear tissue responsible for maintaining endolymph ionic concentrations as well as generating the endocochlear potential (EP). With age, EP cell health and ion levels start to decline due to cellular degeneration in the SV, which compromises auditory function. Considering this, qPCR experiments were performed with SV lateral wall cells extracted from the cochlea of each animal in the hormone groups post‐treatment (in vivo) and in SVK‐1 cells (epithelial cells derived from the SV of the P14 Immortomouse) treated with HRT over various lengths of time (in vitro) to evaluate the expression levels of IGF‐1R and FoxO3. The findings from this study will indicate whether there is cross talk between E, IGF‐1R, and FoxO3 via the PI3K/AKT pathway, which contributes to the delayed onset of ARHL observed during HRT with E in vivo.

Novel aspects of the present study include (a) treating animals with hormone therapy over an extended period of 6 months, (b) administering P alone to evaluate its sole effect on auditory system processing, (c) evaluating the aftermath of HRT on the aging cochlea, and (d) examining the relationships between the IGF‐1 pathway and the protective properties of E. The discoveries from this experimentation will help to gain better insights as to how E preserves cellular functionality in the aging auditory system via neuro‐protective properties, which could lead to future enhancements in therapeutic solutions for preventing or treating key aspects of age‐related hearing loss (ARHL).

## 2 RESULTS

For the present study, CBA/CaJ middle‐aged mice underwent baseline auditory brainstem response (ABR) and ABR gap‐in‐noise (GIN) electrophysiological testing at 15 months of age. All of the female animals underwent an ovariectomy procedure, where both sets of ovaries were removed, at the outset of the study, once baseline hearing testing was complete. This was done to ensure that naturally occurring sex hormones would not add variability to the female hormone levels during mouse “menopause/estropause.” The females were then randomly placed in hormone treatment groups: E‐estradiol 17β (*n* = 16, 0.006 mg/day), P (*n* = 12, 0.40 mg/day), E + P (*n* = 12, 0.40 mg/day +0.006 mg/day), and placebo (Pb, *n* = 13). The hormone treatments utilized for the following investigation were optimal HRT concentrations obtained from previous studies by Guimaraes et al. (2006) and Price et al. (2009). It should be noted that a group of males, also 15 months old at study onset, served as a comparison control group. The animals went through hormone treatment for a duration of 6 months. Subsequently, a 1‐month washout/recovery period was followed to see whether the effects of the hormones were ongoing or reversible.

**Figure 1:**
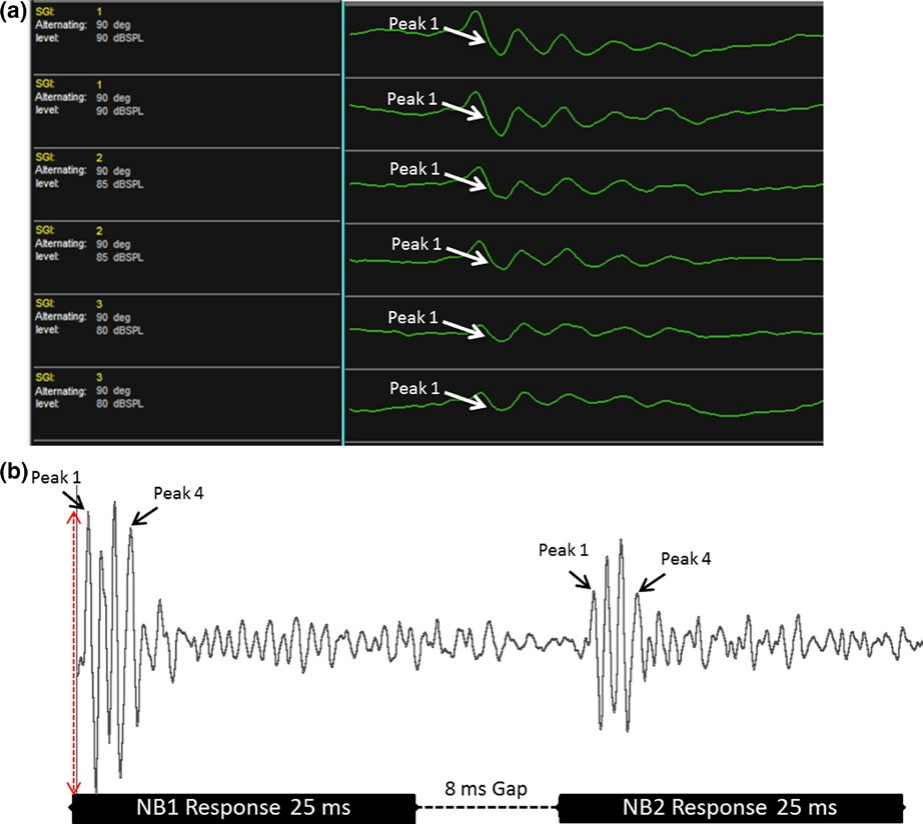
Auditory brainstem response (ABR) and ABR gap‐in‐noise techniques. (a) Five to seven waves are typically generated during Auditory Brainstem Response (ABR) testing for humans clinically, and animals such as mice. Starting at 90 decibels (dB), various frequencies (3–48 kHz) are presented at sound levels that decrease in intervals of 5 dB. The threshold is considered the lowest sound level at which a waveform can be detected. The figure shows the ABR results for a 15‐month‐old CBA/CaJ mouse at 90 decibels (dB). (b) The ABR gap‐in‐noise (GIN) technique measures auditory temporal processing. Subjects were presented with a 25‐millisecond (ms) noise burst (NB1) followed by a series of silent gap durations, ranging from 0 to 64 ms. A second 25‐ms noise bursts (NB2) was presented to measure the ability of the auditory system to recover from NB1 and efficiently respond to NB2. For the present study, the ABR GIN analysis focused on the amplitude levels (red arrow) for Peak 1 and Peak 4. The figure displays the baseline ABR GIN response at 8 ms for a 15‐month‐old mouse

Auditory brainstem response (ABR) tests evaluate not only the cochlear output (Peak 1: P1) but also the brainstem pathways that subserve hearing as well. A short duration sound is presented to the ear of the animal and an EEG waveform response is generated. Each peak of this waveform response is produced from a particular level or nucleus of the auditory brainstem. Specifically for ABRs, P1 is used to determine the threshold for tone pip frequencies ranging from 3 to 48 kHz as well as a wideband noise (WBN) stimulus (Figure 1a). The lowest sound level, in decibels (dB), where P1 can be identified is recognized as the threshold. A novel paradigm known as ABR gap‐in‐noise (GIN) was used to test auditory temporal processing. The ABR GIN technique uses the same basic sounds that elicit ABRs to measure the temporal processing abilities of subjects at various sound gap intervals (Figure 1b). P1 and P4 amplitude levels measure the intensity as well the synchronization of neurons from the auditory nerve and inferior colliculus, respectively, when a noise stimulus has been presented (Williamson, Zhu, Walton, & Frisina, 2015). These hearing tests allowed us to gain better insights into the various stages of the aging process in the auditory system, using declining amplitude levels and high thresholds as an indicator of presbycusis. Further details and explanations about ABRs and ABR GIN testing techniques can be found in our previous, detailed report by Williamson et al. (2015).

### 2.1 Hormonal influences on absolute sensitivity of hearing

**Figure 2:**
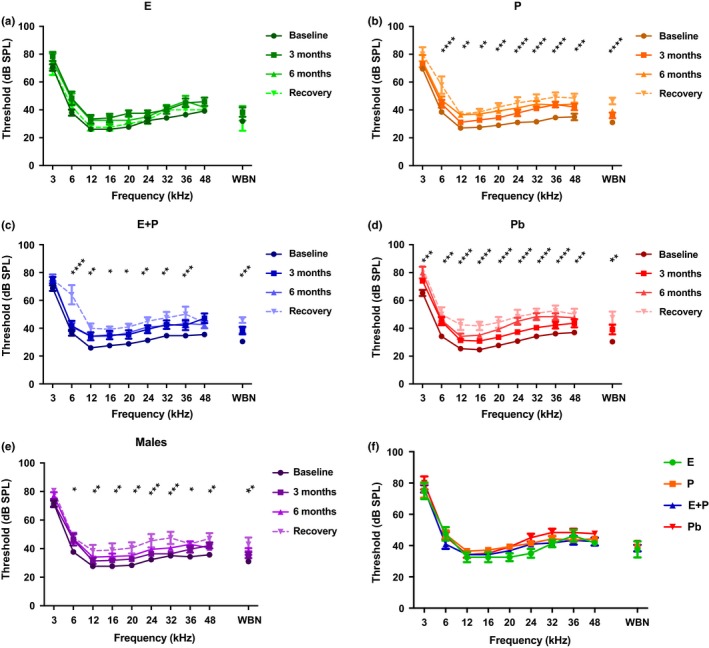
Auditory brainstem response thresholds over the course of hormone treatments as well as during the recovery period. (a) E animals show no significant signs of ARHL over the course of hormone therapy, thus indicating that E possesses protective properties for auditory function. (b) P animals show significantly poorer hearing at almost all of the tested frequencies. (c) The E + P group displayed elevations in ABR thresholds as early as 3 months. Notable worsening of hearing could be seen in this group over time. (d) Pb control animals' thresholds changed drastically over the 6‐month time period. Significant ARHL changes were observed for all frequencies. (e) Changes observed in the male group, more specifically during the recovery period (8 months), could be attributed to ARHL. (f) Recovery period group comparison shows that E‐treated animals had lower thresholds at 12, 16, 20, 24, and 32 kHz compared to all the other HRT animals. Pb females had higher thresholds among the HRT groups at 24 and 32 kHz. These data, in conjunction with Figure 5, suggest that the results of long‐term HRT on ABR thresholds are permanent. No statistical differences were seen among the hormone groups during the recovery period. It should be noted that statistical differences for (a) through (e) are a comparison between the baseline and recovery. Statistical test: 2‐way ANOVA followed by Bonferroni; *p < 0.05, **p < 0.01, ***p < 0.001, ****p < 0.0001

Averaged ABR thresholds in Figure 2 depict the changes that each of the hormone groups underwent during HRT over time (*F*_Time_ [9, 208] = 21.31, *p* < 0.0001). Very few changes were seen in the E animal thresholds over the treatment period compared to the other groups (Figure 2). Statistical differences were only seen at 6 and 20 kHz between the baseline and 3‐month checkpoints (Refer to Supporting Information Table S1, Datasheet). Meanwhile, E + P‐treated animals displayed significant increases in ABR thresholds during HRT as illustrated in Figure 2c. Indeed, differences were seen once treatment ended, especially at 6 kHz, 36 kHz, and for WBN. Interestingly, P and Pb animals had the worst hearing thresholds among all of the hormone groups during the 6 months of treatment as well as during the 1‐month recovery period. The P group's thresholds began to significantly increase as early as 3 months after treatment began, especially for high frequencies (Figure 2b). Supporting Information Table S1 shows a notable increase in the ABR thresholds at 36 and 48 kHz at the 3‐month checkpoint. By 6 months, almost all of the thresholds rose significantly for all of the tested frequencies, relative to the pretreatment baseline. Post‐treatment ABR thresholds were higher than thresholds during treatment and significantly so compared to baseline thresholds. Similarly, Pb animals' auditory function worsened over time during the experiment. Escalated thresholds were observed 3‐month postovariectomy surgery and continued to rise for the remainder of the experiment (Figure 2d). Threshold shifts for this particular group were as high as 10 dB for the higher frequencies, thus indicating that the loss of E without receiving HRT had a substantial impact on Pb animals. ABR thresholds for the male group displayed age‐linked threshold elevations throughout the experiment (Figure 2e). The considerable differences seen in the recovery/washout period, similar to the E + P group, can be attributed to the progression of ARHL for this male control group. Figure 2f shows a comparison all of the hormone groups post‐treatment (*F*_HRT_ [4,241] = 6.215, *p* < 0.0001). No significant differences were found among the groups at the checkpoint; however, the thresholds for the E animals were slightly lower than all of the other groups, specifically at 12, 16, 20, and 24 kHz.

### 2.2 Hormonal influences on changes in auditory temporal processing

**Figure 3:**
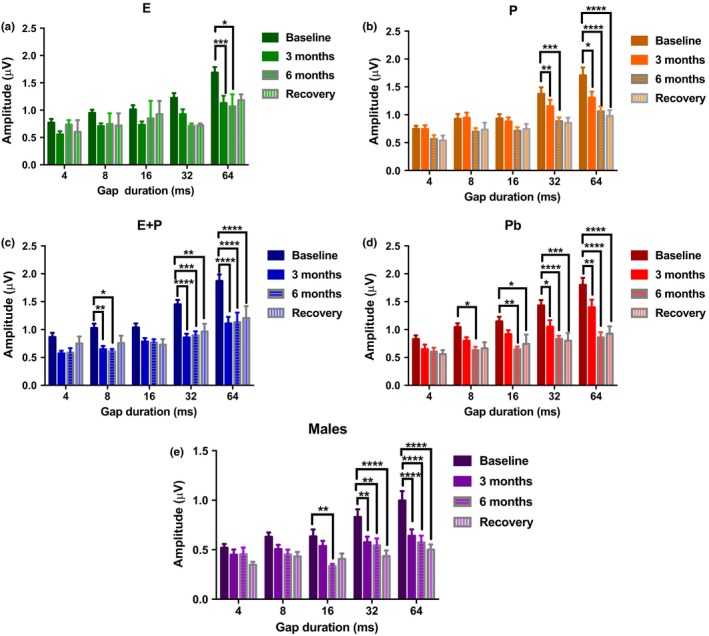
Auditory brainstem response gap‐in‐noise P1 amplitude levels for NB2 for subject groups during HRT and for the recovery period. (a) Few changes were observed in E‐treated animals during the course of the longitudinal experiment. Significant changes were only seen at the largest gap interval, 64 ms. (b) The P group displayed greater declines while undergoing long‐term HRT. (c) Amplitude levels were sharply reduced once treatment began for E + P animals. Significant changes were seen as early as 3 months at 8, 32, and 64 ms. (d) ARHL was observed in Pb animals throughout the course of the experiment. These changes could have been exacerbated due to the removal of E from the circulatory system during middle age. (e) The males also displayed signs of presbycusis throughout the course of the experiment, as steep declines were detected for P1 amplitude levels. These findings suggest that females treated with E or P retain temporal processing abilities better than males. No signs of recovery were observed in any of the subject groups. Statistical test: 2‐way ANOVA followed by Bonferroni; *p < 0.05, **p < 0.01, ***p < 0.001, ****p < 0.0001

Longitudinal ABR GIN amplitude values for P1 were compared for each of the HRT groups, as seen in Figure 3. Similar to ABR thresholds, E‐treated animals had P1 amplitude levels that minimally declined over time compared to the other subject groups. For instance, P1 amplitudes ranged from 1.01 mV to 0.85 mV at 16 ms for the baseline and 6‐month checkpoint, respectively. Notable changes were only seen at 64 ms for 3 and 6 months, as seen in Figure 3a. Interestingly, the P group's amplitude levels exhibited a gradual decline as treatment progressed. For instance, amplitude levels dwindled from 1.71 mV to 1.49 mV at 64 ms for the baseline compared to the 3‐month checkpoint. After 6 months of treatment, this value continued to decrease by 0.43 mV, relative to the 3‐month value. For this particular group, meaningful differences were seen in Figure 3b at wider gap durations: 32 and 64 ms. Recovery amplitude values for these animals were relatively similar to 6‐month values, which suggests that there is *no recovery* from P treatment. As presented in Figure 3c, E + P P1 amplitude levels declined radically by 3 months of treatment. Baseline values that started at 1.87 mV were abruptly reduced to 1.11 mV, 3 months after HRT began, for the 64‐ms gap interval. This decline remained reasonably consistent throughout the course of treatment. Significant differences were seen in gap durations of 8, 32, and 64 ms. No recovery was displayed for this group. Contrary to what was seen with ABR thresholds, E + P had more of a negative effect on ABR GIN amplitude levels than P. Additionally, differences for P1 amplitude levels in female mice were seen in the Pb group. Gradual decreasing values were observed at the smaller gap durations (Figure 3d). Nonetheless, considerable declines were seen as early as 3 months by 32 ms. The male group exhibited a sharp reduction in P1 amplitude levels for each of the gap intervals as well. Parallel to Pb animals, statistical differences were seen at 6 months into the experiment for smaller gap durations; meanwhile, changes were seen as early as 3 months at 32 and 64 ms (Figure 3e). The fact that the Pb and the male groups displayed similar trends in P1 amplitude values imply that ARHL‐induced auditory temporal processing deficits are occurring in the mice at middle age—18 months. None of the animal hormone groups had P1 amplitude levels that reverted back (or close) to baseline values during the recovery period; therefore, the effects of long‐term HRT are indeed permanent.

**Figure 4:**
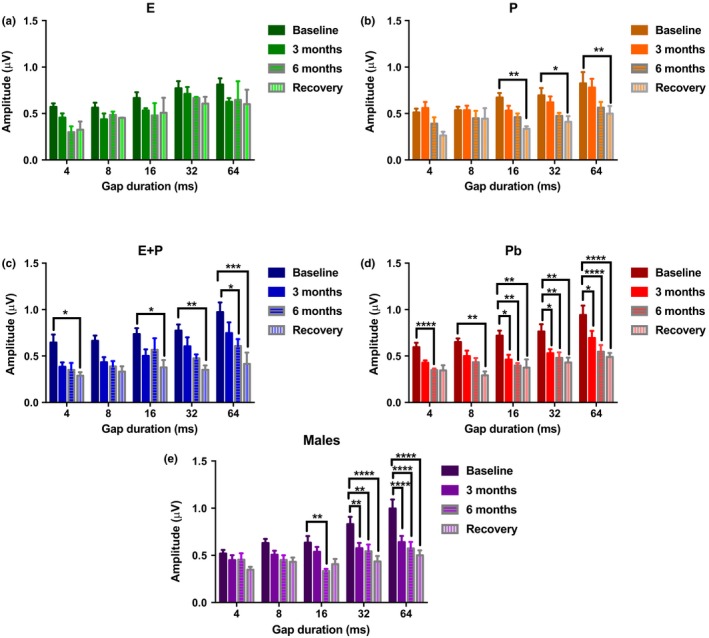
Auditory brainstem response gap‐in‐noise P4 amplitude levels for NB2 for subject groups during HRT and for the recovery period. (a) Amplitude levels for E‐treated animals decline marginally once treatment began. (b) The P group amplitude levels showed a steady decline throughout the advancement of treatment, with the largest differences seen during the recovery period. (c) E + P animals also displayed a significant decrease in P4 amplitude levels. Most striking differences were observed during the recovery period. (d) Amplitude values declined as early as 3 months for Pb animals. This group exhibited the worst reduction in amplitude levels among the female groups. (e) The males also showed signs of ARHL at longer gap intervals, starting at 16 ms. All of the groups, except E displayed changes during recovery, that is, for these groups P4 amplitudes worsened after HRT was discontinued. Statistical test: 2‐way ANOVA followed by Bonferroni; *p < 0.05, **p < 0.01, ***p < 0.001, ****p < 0.0001

P4 amplitude levels for NB2 in Figure 4 illustrate various changes among the HRT groups. E‐treated animals had relatively stable amplitude levels while undergoing HRT and during the recovery period. Thus, no statistical differences were observed for the E group in Figure 4a. P animals displayed P4 amplitude values that progressively worsened throughout the experiment. For example, baseline P4 amplitude level of 0.82 mV declined by 0.26 mV at 6 months into hormone therapy for 64‐ms gap intervals (Figure 4b). This value dropped to 0.5 mV by the end of the recovery/washout period. Meaningful changes were seen at 16, 32, and 64 ms for recovery. Compared to P1 amplitude levels, P4 amplitude values continued to decrease during the recovery period for the P group. This indicates that P4 amplitudes showed preferential worsening, relative to P1, after treatments were discontinued. This suggests relatively negative effects on the auditory brainstem. E + P proved to be detrimental to auditory function as displayed in Figure 4c. P4 amplitude levels drastically diminished, especially at longer gap intervals. At 64 ms, the average baseline level of 0.97 mV dropped to 0.42 mV during the recovery period. Similar to P animals, amplitudes declined during the recovery period for all of the gap durations, suggesting that E + P worsens temporal processing abilities for P4 even after treatment has ended. Pb animals displayed the most rapid regression in amplitude levels among the female groups. Notable variations were observed at all of the gap intervals, particularly for gaps of 16 ms or longer, as seen in Figure 4d. Intriguingly, most of the recovery amplitude levels resembled those values seen at the 6‐month checkpoint. Lastly, the male group displayed fewer age‐related changes than the Pb group for P4 amplitude levels at the shorter gap durations, but just as severe declines for the longer gaps. Specifically, significant changes occurred at 3 months for 32 and 62 ms (Figure 4e). Similar to Pb, these changes are strong indications of brainstem temporal processing deficits characteristic of ARHL. However, in this case, the males show smaller declines at the shorter gap durations than the E + P and Pb females.

### 2.3 In Vitro SVK‐1 cells display increases in IGF‐1R expression during hormone treatment

**Figure 5:**
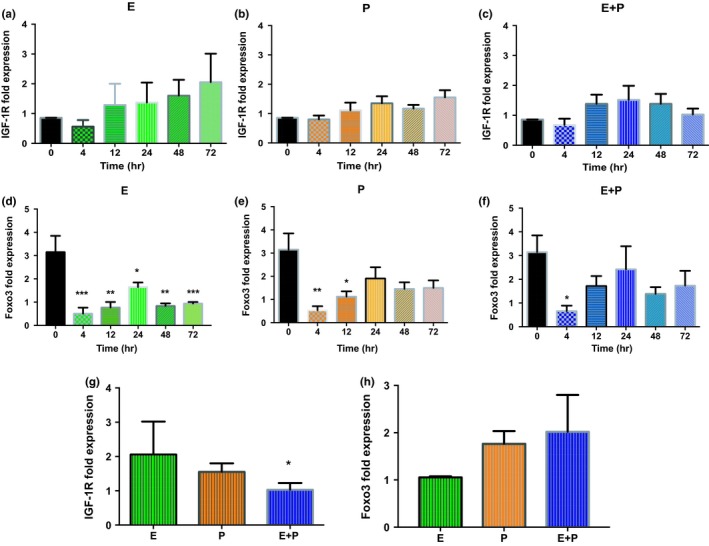
In vitro quantitative IGF‐1R and FoxO3 gene expression in SVK‐1 cells after different time intervals for the duration of HRT. (a) IGF‐1R gene expression displayed an upward trend once E treatment began. Most notably, this expression more than doubled after 72 hr of E hormone therapy. Contrastingly, (b) P‐ and (c) E + P‐treated cells had IGF‐1R expression levels that were relatively the same over the course of the experiment, suggesting that these hormone treatments had little to no effect. (d) E‐treated cells exhibited FoxO3 levels that significantly declined. Statistical differences were observed throughout the course of HRT. (e) P, and (f) E + P displayed similar trends in which FoxO3 expression levels dramatically decreased after 4 hr of treatment; however, gene levels began to somewhat increase, especially by 24 hr. The statistical differences are relative to the untreated cells at 0 hr. Statistical test: 1‐way ANOVA followed by Bonferroni; *p < 0.05, **p < 0.01, ***p < 0.001. Comparison among the hormone‐treated cell groups after 72 hr of HRT for (g) IGF‐1R and (h) FoxO3 gene expression. (g) E maintained high IGF‐1R levels over time; meanwhile, E + P cells displayed gene expression levels that continued to decline at 72 hr, causing this cell group to have the lowest expression. A significant difference was observed for the E + P group relative to the E group. (h) Although E had FoxO3 expression levels that were lower than both P and E + P SVK‐1 cells, no significant differences were observed among the groups at 72 hr. Statistical test: Welch's *t* test followed by Bonferroni; *p < 0.033

A focal purpose of the present study was to investigate key properties of the PI3k/AKT pathway and its role in cochlear cell survival as well as anti‐apoptotic responses, which can be regulated by IGF‐1R and FoxO3 genes. In vitro studies were performed to observe the expression of IGF‐1R and FoxO3 during the course of HRT to better interpret the findings from electrophysiology experiments. SVK‐1 cells were treated with E, P, or E + P and were evaluated in a time‐dependent manner: 4, 12, 24, 48, and 72 hr. (Untreated cells are denoted at 0 hr and served as the control.) E‐treated cells were the only group to demonstrate an increasing trend with time for IGF‐1 levels, as seen in Figure 5a. Fold change expressions that were initially 0.56 at 4 hr rose to 2.21 toward 72 hr of E treatment, which is more than twice the starting value. It should be noted that IGF‐1 gene expression *marginally*declined, relative to untreated cells (0.86), during the first few hours of treatment before the positive trend was observed. In contrast, SVK‐1 cells treated with P and E + P exhibited little to no changes in expression during hormone therapy in Figure 5b,c, respectively. For instance, at 4 hr E + P cells had gene expression levels of 0.68 that elevated to 1.5 at 24 hr and then dwindled to 1.02 at 72 hr. These findings indicate that over a long‐term period E could possibly play a key role in amplifying the expression of IGF‐1 in the cochlea. Meanwhile, P and combination treatment (E + P) showed no effect, which may have contributed to functional declines and degeneration in the auditory system observed during the electrophysiology experiments.

FoxO3 gene expression was also evaluated in hormone‐treated SVK‐1 cells, as seen in Figure 5 (d–f, h). Untreated cells had an average FoxO3 expression value of 3.14. Intriguingly, all of the HRT cell groups had FoxO3 expression levels that decreased significantly immediately after treatment began; however, only E‐treated cells displayed statistical differences over the entire time course of hormone therapy (Figure 5d). More specifically, SVK‐1 cells undergoing E therapy had FoxO3 levels that were reduced to a value of 0.51 at 4 hr. The gene expression gradually changed to 1.64 by 24 hr, which is about half of the untreated cell's FoxO3 expression value; 48 hr later, gene levels began to decline once again, reducing the value to 0.93 by the end of the experiment. This similar pattern was observed less dramatically in P‐ and E + P‐treated cells. As presented in Figure 5e, SVK‐1 cells treated with P had FoxO3 levels that notably declined in a span of 12 hr. Similarly, E + P cells displayed a downtrend gene expression value of 0.66 at 4 hr (Figure 5f). These results strongly suggest that E and, to a lesser extent, P modify FoxO3 levels in the cochlea. However, the reason for the variability in the gene expression at the different time points is not fully understood. There is a possibility that E treatment causes a downtrend in FoxO3 gene levels, seeing that at 72 hr of treatment this was the only group that had diminishing expression values. Interestingly, P and E + P had higher FoxO3 expression levels among the cell groups throughout the experiment, with values of 1.5 and 1.73, correspondingly (Figure 5e,f,h). This indicates that E not only maintains lower FoxO3 levels in the auditory system over time, but does so more effectively than P. This reduction in FoxO3 expression may activate another component in the PI3K/AKT pathway to assist cochlear cells with neuro‐protective activities. Further testing should be done to determine whether the declines in expression seen during treatment improve or worsen for longer time periods.

### 2.4 In Vivo SV tissue samples exhibit lingering effects post‐treatment

**Figure 6:**
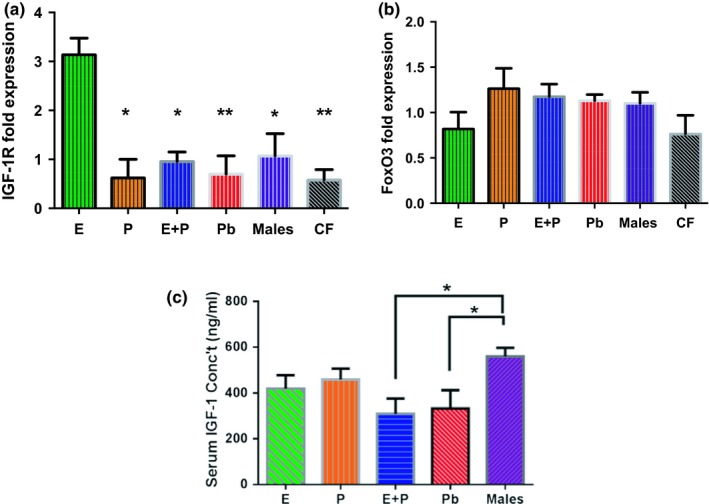
In vivo 1‐month post‐treatment IGF‐1R and FoxO3 expression levels. (a) E animals had the highest IGF‐1R fold expression levels among the subject groups for SV tissue samples. Interestingly, Pb and control female (CF) animals had the most significant differences among the groups, relative to E. This implies that lack of HRT during the aging process could possibly decrease IGF‐1R levels. (b) FoxO3 gene expression was comparatively similar among the SV tissue sample groups. Congruous findings were observed for in vitro FoxO3 experiments. It can be noted that overall the CF group had the lowest expression levels for both genes. (c) Post‐treatment IGF‐1 concentration levels in the serum of HRT mice showed no significant differences among the female HRT groups. Only E + P and Pb groups displayed statistical variances in comparison to the control male animals. Statistical test: 1‐way ANOVA followed by Bonferroni; *p < 0.05, **p < 0.01 (E *n* = 3; P *n* = 3; E + P *n* = 3; Pb *n* = 3, Males *n* = 3; CF *n* = 3). *Note*: The CF group consists of age‐matched females with their ovaries intact that did not undergo any type of HRT

In vivo SV tissue samples were utilized to evaluate the 1‐month aftermath effects of long‐term HRT in the peripheral auditory system. These results were also used to validate the findings obtained from molecular experiments using SVK‐1 cells (this section). Figure 6 shows that SV tissue samples from E‐treated animals had *significantly*higher IGF‐1R levels, by approximately threefold, compared to the rest of the groups in the study (*F*_HRT_ [5,10] = 6.50, *p* = 0.0061). The Pb and control female (CF; age‐matched females with ovaries intact) groups had the most significant differences, relative to the E group, among the other subject groups. This is an interesting finding due to the fact that neither of these female groups underwent any type of hormone treatment. Therefore, it is possible that lack of HRT, during the aging process, decreases IGF‐1R levels. Conversely, FoxO3 expressions were relatively similar among all of the groups, with expression levels ranging from 0.76 (CF) to 1.26 (P) (*F*_HRT_ [5,11] = 1.43, *p* = 0.29). It is important to note that these are the gene expression values 1 month after HRT was discontinued.

In Figure 5g and 6a, e‐treated cells displayed higher levels of IGF‐1R *both*during and after HRT. This coincides with previous reports of E's long‐lasting effect on the auditory system. E + P‐treated cells had the lowest IGF‐1R expression among the hormone groups during the treatment period. Although E‐treated cells presented somewhat of a lower trend for FoxO3 levels compared to E + P SVK‐1 cells, no significant changes were observed after 72 hr of hormone therapy for any of the groups (Figure 5h). Parallel results could be seen among the hormone groups for in vitro and in vivo IGF‐1R and FoxO3 gene expressions. These findings suggest the theory that IGF‐1R and FoxO3 may have an inverse relationship with one another during sex hormone therapy.

Additionally, serum IGF‐1 expression levels were taken from animals that underwent HRT for the duration of 6 months, followed by the 1‐month recovery period, which are shown in Figure 6c. Significant differences were only seen between the E + P, Pb, and male control groups. These findings indicate that hormones have somewhat of an impact on IGF‐1 levels, albeit no significant differences were observed for any of the females.

## 3 DISCUSSION

### 3.1 E's Neuro‐protective properties in the cochlea

Our study showed positive findings for E‐treated animals that can be used to expand on the theory that this hormone has neuro‐protective properties in the auditory system that could possibly prevent or delay key aspects of presbycusis. As presented above, the E group displayed minimal changes in ABR thresholds while undergoing HRT (Figure 2a). Additionally, this group of animals consistently had lower auditory thresholds during the recovery/washout period than all of the other HRT groups (Refer to Figure 2f). Price et al. (2009) observed similar results with ABR thresholds in middle‐aged female mice treated with E for 4 months compared to age‐matched mice treated with E + P for the same amount of time and their male control group. It should be noted that the ovaries for the CBA/CaJ female mice used in the Price et al. (2009) study were intact throughout the course of the treatment. Similarly, postmenopausal women treated with E for a mean of 3.35 ± 2.20 years had consistently lower air conduction thresholds than women who received no treatment and women who were undergoing combination hormone therapy for 4.13 ± 2.41 years (Kilicdag et al., 2004). As for P1 and P4 amplitude levels for gap coding experiments (NB2, in the present study), minimal changes were also observed. P1 amplitude values for 3 months, 6 months, and recovery were essentially the same for the E group at a majority of the gap durations (Figure 3a). Likewise, P4 amplitude levels remained relatively stable once hormone therapy began. These findings support Coleman and colleagues (1994) work with 3‐month ovariectomized (OVX) rats treated with E that maintained high amplitude levels compared to OVX rats that did not receive any treatment. Analogously, Wharton and Church (1990) observed declining ABR amplitude values in young women (19–25 years of age) relative to old females (50–75 years of age). Young women had amplitude values of 0.41 mV that drastically declined to 0.3 mV in older women for ABR Wave V (P4 mouse equivalent) at 80 dB. Contrarily, their young and old male counterparts had amplitudes with a difference of only 0.01 mV for Wave V at 80 dB. This steep age‐related decline in female amplitude levels in the Wharton and Church (1990) study was more than likely due to the decrease of E in postmenopausal women aged 50+.

These lines of research have come a long way in explaining E's protective benefits for the peripheral and central auditory systems (Frisina & Frisina, 2013; Jerger & Hall, 1980). To date, there are several theories as to how E preserves auditory function both directly and indirectly. The leading theory is that E assists in the regulation of neuron survival, which is an important neurotrophic component that is lost during the aging process. Estrogen receptor (ER) subtypes, ERα and ERβ, have been identified in the inner ear, in areas such as the stria vascularis (SV), cochlear blood vessels, and spiral ganglion (SG) type I cells. ERα has been linked to alterations of cochlear and vestibular sensory transduction; meanwhile, ERβ is associated with the survival of neurons in the auditory system (Garcia‐Segura, Azcoitia, & DonCarlos, 2001; He & Ren, 2018; Meltser et al., 2008; Motohashi et al., 2010). From this knowledge base, it has been proposed that ERβ could possibly play a significant role in the ascending auditory pathway in transmitting information from the cochlea to the brain more effectively. It is well known that for hearing transduction, sound waves are converted into electrochemical signals via the inner hair cells (IHCs). IHCs synapse primarily with type I SG cells, exciting the auditory nerve, which in return relays sound information to the central auditory regions of the brain. The synchronization and number of SG cells that respond to a sound stimulus determine the amplitude and latency of ABR waves (Williamson et al., 2015). Therefore, attenuating amplitude levels and increasing latency values are correlated with ERβ degeneration in the auditory system (Charitidi & Canlon, 2010; Charitidi, Meltser, Tahera, & Canlon, 2009; Stenberg, Simonoska, Stygar, Sahlin, & Hultcrantz, 2003; Stenberg et al., 2001). Previous studies that support this theory include Hultcrantz, Simonoska, and Stenberg (2006) who found that ERβ knockout mice displayed rapid declines in auditory function as early as 12 months, which eventually led to severe degeneration throughout the parts of the brain used for hearing after 1 year. Furthermore, Rudzinski and Krejza (2002) observed that E ligand interactions with ERs increase with the up‐regulation of growth factors and specific genes (IGF‐1 and FoxO3) responsible for cell proliferation, metabolism, and anti‐apoptotic cellular responses. Therefore, it is possible that steady levels of E help to keep ER's intact, which could delay the degeneration of cochlear cells and auditory neurons. The molecular pathway findings for E therapy of the present report also strongly support these conclusions.

The findings from this study imply that E may have a beneficial effect on IGF‐1R expression in the cochlea since E‐treated SVK‐1 cells displayed an upward trend for IGF‐1R levels during a 72‐hr time span (Figure 5a). Even more interesting was the in vivo finding that IGF‐1R expression levels were significantly high 1‐month post*‐*treatment in SV samples extracted from the cochlea of E mice compared to the rest of the subject groups (Figure 6a). These results support the growing evidence that E and IGF‐1R may have a co‐dependent relationship with one another in the aging cochlea.

Recent findings from other physiological systems support the growing evidence that E has a positive relationship with IGF‐1R, in order to manage oxidative stress and promote cell survival (Rodriguez‐de la Rosa, Lassaletta, Calvino, Murillo‐Cuesta, & Varela‐Nieto, 2017; Sohrabji, 2015). Olivieri et al. (2014) found that IGF‐1R expression levels doubled in MCF‐7 cells when E dosages were raised from 10 to 100 nM. This increased expression of IGF‐1R proved to have beneficial value on cell signaling, mobility, and muscle power in the skeletal muscle of postmenopausal women undergoing HRT. This study concluded that E increases activity in the IGF‐1 pathway, significantly delaying the atrophy of skeletal muscle in HRT users. Therefore, the results of the present study can be interpreted commensurately: High IGF‐1R expression levels that were observed in E‐treated SVK‐1 cells were protecting cochlear sensory cells from degeneration. This is further supported by the fact that various studies have found that E improves and maintains auditory function via its neuro‐protective properties. As mentioned above, one of the main theories for how this occurs is ERβ association with the survival of cochlear cells (Hultcrantz et al., 2006; Motohashi et al., 2010; Stenberg et al., 2003). Perhaps there is cross talk specifically between ERβ and IGF‐1R pathways in the auditory system. A pioneering study carried out by Toran‐Allerand et al. (1988) showed that cultures of the olfactory bulb, hypothalamus, preoptic area, and cerebral cortex treated with E and high levels of insulin exhibited significant increases in neurite outgrowth. This increase in neuronal growth was limited to areas that only had ERs. Quesada et al. (2007) found that SNpc DA neurons found in the brain were immunoreactive for ERβ and IGF‐1R, which helps to explain why E's neuro‐protective effects are related to IGF‐1. It should be noted that ERα was not identified in this particular neuron. Furthermore, primary cortical neurons treated with the excitatory neurotransmitter glutamate to induce neuronal death, exhibited signs of neuro‐protection with decreased cellular lactate dehydrogenase (LDH) levels after being administered E. (Singer, Rogers, Strickland, & Dorsa, 1996). LDH levels increased in pretreated E cells given Tamoxifen (the major blocker of ERs used clinically) over a 24‐hr period, reversing any signs of neuro‐protection.

The fact that E activates many biological events leads to a number of possibilities of which downstream pathways are involved in this neuro‐protection in the auditory system. In the present study, we hypothesized that AKT phosphorylated by IGF‐1 would activate FoxO3 gene expression, which would inhibit the DNA transcription of pro‐apoptotic genes (i.e., BIM, Fas ligand) via the PI3K/AKT pathway (Kops et al., 2002). In response, cochlear cells would be safeguarded from apoptosis and the signs of ARHL would be delayed. Previous studies have found that forkhead transcription factor, FoxO3, plays a role in preserving auditory function (Gilels, Paquette, Beaulac, Bullen, & White, 2017; Gilels, Paquette, Zhang, Rahman, & White, 2013). Nonetheless, FoxO3 gene expressions statistically fell from 3.14 to 0.51 after only 4 hr of E treatment (Refer to Figure 5d). These levels suddenly increased after 12 and 24 hr; however, FoxO3 gene expression values began to dwindle at 48 hr. Similar trends could be seen among all of the hormone groups in Figure 5 d‐f. By 72 hr, the E group had a downtrend of FoxO3 levels, especially compared to E + P cells. Parallel results could be seen with in vivo post‐treatment experiments (Figure 6b). Surprisingly, the up/down inclination of FoxO3 levels observed in SVK‐1 cells did not mirror the results of the serum levels obtained during HRT in vivo experiments. That does not mean, however, that changes did not take place in cochlear cells during treatment. FoxO3 is heavily regulated by the phosphorylation of AKT based upon environmental conditions. Untreated SVK‐1 cells had qPCR values of 0.86 and 3.14 for IGF‐1R and FoxO3 expression levels, respectively. All of the cells that were administered E had FoxO3 levels that displayed lower expression values than control cells, most significantly at 72 hr. IGF‐1R levels, on the other hand, displayed a continually upward trend for E‐treated cells over the course of the experiment. It is possible that the upward proclivity of IGF‐1R led to the downtrend of FoxO3 in the cochlea, as a concurrent response. For instance, a study that was performed using MCF‐7 cells discovered that IGF‐1R expression increased with incremental dosages of E; however, FoxO3 levels remained the same after 10 and 100 nM of E treatment (Olivieri et al., 2014). It is possible that the vast increase of IGF‐1R in SVK‐1 cells could have activated another signaling pathway to prevent apoptosis from occurring without the need to accumulate FoxO3. This suggests that FoxO3 is not a primary gene that induces survival in cochlear cells in the IGF‐1 pathway during HRT (Zekas & Prossnitz, 2015). More research is needed to confirm which related cell signaling genes are activated by IGF‐1R to stimulate the PI3K/AKT pathway to prevent apoptosis.

### 3.2 P's controversial impact on auditory functionality

Initially, P seemed to have a negative impact on OVX mice as seen in Figure 2b. As soon as treatment began, ABR thresholds increased drastically by 10 dB for high frequencies at 3 months. This escalation continued throughout the course of hormone therapy. By recovery time, thresholds shifts were approximately 20 dB for higher frequencies, relative to the baseline. No recovery was observed. Interestingly, ABR GIN amplitude values for P1 and P4 for NB2 gradually declined for P‐treated animals. For smaller gap durations, baseline values and 3‐month values were comparatively the same for P1. Signs of auditory deterioration were present after 6 months of treatment. Significant differences can be seen for 6 months and recovery for wider gap intervals, such as 32 and 64 ms. A similar trend could be seen with P4 amplitude values, but significant differences were seen only at recovery. Once treatment ended, P4 amplitudes dropped to nearly 50% compared to baseline values. This is an indication that P exhibits some type of protection for temporal processing in the brainstem (lateral lemniscus/IC), since P4 waves are believed to be generated from that region of the brain (Williamson et al., 2015). It should be noted that P's overall effects on the auditory system still remain unclear. Very few hormone‐based studies have evaluated auditory function for subject groups treated with only P. Possibly because researchers surmise that P's effects on the auditory system are indirect (Bonnard, Sahlin, Hultcrantz, & Simonoska, 2013). It has been speculated that since E has such a positive impact on the auditory system, P must be the negative component of the hormone duo, E + P. For instance, E + P has been shown to be detrimental to hearing thresholds in various human and animal studies (Guimaraes et al., 2006; Kilicdag et al., 2004; Price et al., 2009). This theory includes the notion that P acts as an inhibitor to balance out the excitatory, neurotrophic effects of E. For instance, high levels of P and its metabolites can potentially activate the synthesis of an inhibitory neurotransmitter known as GABA, which leads to an increase in inhibitory synaptic transmission in the brain and the cochlea, disrupting the normal balance of excitatory and inhibitory drives (Guimaraes et al., 2006; Rogawski, 2003). This rise in inhibition may degrade auditory threshold sensitivity and supra‐threshold responses. Contrarily, E is believed to have the opposite effect, by decreasing GABA levels in the brain and cochlea to more favorable amounts (Ledoux & Woolley, 2005). This logic can explain the negative impact that P had on ABR thresholds for the present study (Figure 1b). However, favorable auditory responses were observed for the ABR GIN amplitude levels for P‐treated animals. Therefore, although P is not as consistently beneficial as E, under certain circumstances P alone may possibly possess some neuro‐protection properties in the auditory system. This theory is somewhat congruous with the findings from the in vitro (during treatment) and in vivo (post‐treatment) molecular experiments in the present study. Little to no changes were observed for the IGF‐1R gene expression values for P‐treated SVK‐1 cells (Figure 5b). However, there were no statistical differences between IGF‐1R levels for E‐ and P‐treated cells at 72 hr, unlike the E + P group. FoxO3 levels were notably reduced compared to untreated cells, particularly at 4 and 12 hr of treatment as seen in Figure 5e. Post‐treatment results show that P‐treated animals had relatively similar IGF‐1R and FoxO3 gene expression values (Refer to Figure 6). These data suggest that P may have displayed protective properties for cochlear cells during treatment, similar to the P4 amplitude level results previously mentioned. Consistent with this, several studies have found that P increases neurogenesis and neuron survival in the brain (Chan, Chow, Hamson, Lieblich, & Galea, 2014; He, Yang, Zhai, Shao, & Li, 2011; Si et al., 2013). For instance, male rats with traumatic brain injury (TBI) exhibited significantly lower levels of cyclooxygenase‐2 (inflammation) and caspase‐3 (apoptosis) after being treated with P (Si et al., 2013). Additionally, Berent‐Spillson et al. (2015) reported that postmenopausal women treated with P had the same improvements in verbal processing and verbal working memory as women treated with E, for postmenopausal women treated with HRT for a 90‐day period. It should be noted that verbal processing is related to auditory temporal processing. In another neuroscience report, mice treated with 20 mg of P displayed a 6‐fold reduction in vacuolated motoneurons and a reduction in nitric oxide synthase (NOS) active neurons, both of which are associated with neurodegenerative conditions, including amyotrophic lateral sclerosis (ALS) (De Nicola et al., 2013). These changes improved certain symptoms of this disease in the mice over time.

Although quite a few studies have shown the positive influence of P in the brain after traumatic injuries or neurological diseases, P's impact on the auditory system is still debatable. Our overall findings show that P can have negative impacts on ABRs; however, ABR GIN amplitude levels seem to show P's abilities to possibly preserve certain aspects of auditory system functionality for temporal processing mechanisms. Further investigation needs to be done to gain better insights into P's general role in hearing and aging.

### 3.3 Combination therapy's detrimental influence on hearing

Prior work has shown that combination treatment has a negative impact on the auditory system. In the present study, E + P had a detrimental effect on ABR thresholds compared to E and Pb animals. Threshold values increased by about 10 dB immediately after treatment began, and leveled off during the 6‐month period. ABR Recovery/washout thresholds in Figure 2f show that the E + P group has the second lowest thresholds indicating that this group performed slightly better in ABR's than all of the female hormone groups except for E. P1 ABR GIN amplitude values for E + P animals present a sharp reduction immediately after undergoing HRT. Amplitude levels were cut roughly in half by the 3‐month checkpoint for all of the gap durations, relative to the baseline. For instance, baseline values that started at 1.5 μV were drastically reduced to ~0.8 μV after 3 months at 32 ms. Similar to the ABR thresholds, this decline subsided during the 6‐month and recovery period. These findings suggest that the negative effects of combination treatment occur at a faster pace than compared to P, whose group's amplitude values gradually decreased over the course of the experiment (Figure 3). On the contrary, P4 amplitudes exhibited signs of auditory decline at a steadier rate compared to P1. Significant differences were highlighted between baseline and recovery values. Interestingly, amplitude levels decreased for most of the gap intervals after treatment was discontinued. Similar to the results found with the P group, combination treatment may have had some *marginal*auditory benefits while being administered for P4 but not P1. These findings suggest that E + P may have more of a long‐lasting negative impact on the auditory nerve (P1) as opposed to the IC (P4). Perhaps E + P directly or indirectly affects the auditory nerve fibers. Studies have found that declines in cochlear neurons, seen with aging, can limit the excitatory effects of the auditory nerve during a noise stimulus. Hence, a less than robust P1 will be generated.

Several reports have shown that E + P raises ABR thresholds as well as reduces ABR amplitude level values. Price et al. (2009) presented statistical differences in ABR thresholds and DPOAE amplitudes in E + P postmenopausal mice after 4 months of treatment. This particular subject group was the only group of animals to display notable deficits in DPOAE amplitudes for all frequencies. Similar results were presented by Guimaraes et al. (2006) in post‐menopausal women. In 2001, Bittar and colleagues attempted to explain these types of findings by reporting histological changes of inflammation infiltrate and vacuolization of the SV in guinea pigs treated with E + P. The molecular findings from the present study also suggest that inflammation could be occurring linked to increases in apoptosis. For example, in vitro IGF‐1R gene expression was *significantly* lower for E + P‐treated cells, especially compared to the E group. Meanwhile, FoxO3 gene expression demonstrated an upward trend for E + P cells among the hormone groups, notably at 72 hr (Figure 5h). Even though FoxO3 levels were high for E + P cells, this value was low compared to untreated control cells (Figure 5f). Therefore, FoxO3's ability to inhibit pro‐apoptotic genes and/or remove toxins from the cell via the activation of autophagy‐related genes may have been jeopardized (Vasudevan & Garraway, 2010). This could have prevented the cell from creating a healthy environment to maintain homeostasis and overall integrity; thus, catalyzing the signs of aging within the auditory system. Notably, low IGF‐1R levels could not compensate the protection initially provided by FoxO3, unlike what was observed for E cell group. There are still very few studies that have looked into the negative impact of combination treatment on the auditory system from cellular and biomarker perspectives. Optimistically, these findings will contribute to better understanding how E + P exacerbates ARHL as well as lead to more hormone‐based molecular mechanism investigations in the future.

In conclusion, female sex hormones, E in particular, modified IGF‐1R and, to a lesser extent, FoxO3 expression via the PI3K/AKT pathway in the mammalian cochlea to promote sensory cell health, and delay certain key aspects of ARHL. These effects appear to be *long lasting* in females undergoing hormone treatment. Additionally, P therapy generated conflicting results by both *increasing* ABR thresholds but *slowing* temporal processing deficiencies in ABR GIN amplitude levels associated with aging during the course of HRT treatment. This strongly suggests that P may not be as detrimental to the auditory system as initially thought. Overall, understanding how sex hormones influence auditory function will help menopausal women, who are considering HRT, make more informed decisions that best suits their needs, enhance therapeutic options for ARHL, and possibly lead to more successful research about the relations between hormone levels and other neurodegenerative diseases or dementias, such as Alzheimer's disease, which have been associated with presbycusis.

## 4 METHODS—EXPERIMENTAL PROCEDURES

### 4.1 Animals

For the present study, 70 CBA/CaJ middle‐aged mice were used; 53 females and 17 males. Baseline testing was performed at 15 months on all of the animals. All of the female mice underwent an ovariectomy procedure, where both sets of ovaries were removed, once baseline testing was complete. This was done to ensure that no naturally occurring sex hormones would have any effects. The females were then randomly placed in hormone treatment groups that consisted of E‐estradiol 17β (*n* = 16, 0.006 mg/day), P (*n* = 12, 0.40 mg/day), E + P (*n* = 12, 0.40 mg/day +0.006 mg/day), and placebo (Pb, *n* = 13). It should be noted that a group of males served as a comparison control group. Hormone therapy was administered in the form of a subcutaneous slow‐release pellet. The pellets were designed to release hormones for a duration of 3 months and were inserted between the neck and the shoulder of the animals immediately after the ovariectomy surgery. A second pellet was inserted in the animals after 3 months, ensuring that the females underwent continuous hormone therapy for a total of 6 months. A 1‐month recovery/washout period took place to see whether there were any lingering side effects after HRT was discontinued. CBA/CaJ breeders were obtained from the Jackson Labs (Bar Harbor, ME) and were bred at the University of South Florida (USF) Vivarium. All of the animal protocols used for the following study were approved by the USF Institutional Animal Care and Use Committee (IACUC).

### 4.2 Electrophysiology experiments

Throughout the course of the experiment, the animals hearing was thoroughly tested at different checkpoints: baseline (before surgery and prior to HRT), 3, 6 and 1 month (post‐HRT). Auditory testing consisted of auditory brainstem responses (ABRs) and ABR gap‐in‐noise (GIN). Further details about ABR and ABR GIN testing techniques can be found in our previous, detailed report Williamson et al. (2015). For the animals that completed the study, tissue and blood samples were collected and stored for anatomical and molecular experiments described below.

### 4.3 Cell culture and hormonal treatments

SVK‐1 epithelial cells derived from the SV of the P14 Immortomouse (obtained from Dr. Federico Kalinec, Univ. South. Cal.) were utilized. DMEM medium (Corning, Manassas, VA) with 10% FBS was used to proliferate the cells. Initially, the SVK‐1 cells were incubated at 33°C in a humidified 10% CO_2_ atmosphere in a proliferation stage. At least 24 hr before treatment, the cells were then placed in a differentiation setting of 39°C and 5% CO_2_ to ensure that the cells successfully matured to SVK‐1 epithelial cells in serum‐free DMEM. Once the optimal dosage was determined, the cells received 10 nM of hormone treatment (E, P, or E + P) for various time durations, which included 4, 12, 24, 48, and 72 hr. 10 nM was the optimum dosage for HRT treatment for cells according to previous studies (Duenas, Torres‐Aleman, Naftolin, & Garcia‐Segura, 1996; Olivieri et al., 2014). It should be noted that cells were administered HRT at a confluence of ~80% for treatment durations of 24 hr or less. Hormone therapy lasting more than 24 hr began when a confluence of ~30% was obtained to increase the number of healthy cells that would be collected once treatment ended.

### 4.4 PCR experiments

Following treatment, the cells were washed and extracted using the protocol from the RNeasy Mini Kit (Qiagen). SV tissue samples that were extracted from the cochlea of animals from the HRT ABR study were also used for the following experiment. The samples were placed in RLT Buffer and then extracted using the same protocol as the SVK‐1 cells, previously mentioned. The samples were categorized according to the treatment each animal received: E (*n* = 4, 0.006 mg/day), P (*n* = 4, 0.40 mg/day), E + P (*n* = 4, 0.40 mg/day +0.006 mg/day), and placebo (Pb, *n* = 3). 50 ng for each of the following RNA samples was then used to synthesize 20 µl of cDNA using an iScript cDNA kit (Bio‐Rad Laboratories; Hercules, CA). Once the sample mixtures were made, they were incubated for 5 min at 25°C (priming), 20 min at 46°C (reverse transcription), and 1 min at 95°C (RT inactivation). Primer sequences used to detect the genes were as follows: IGF‐1R, sense 5′‐TTGCCCTAAAACTGAAGCTGA‐3′; anti‐sense 5′‐GTTCTCGCAAAGACGAAGTTG‐3′ and Foxo3, sense 5′‐GTTCAATGGGAGCTTGGAAT‐3′; anti‐sense 5′‐CAACCCGTCAGCATCCATGA‐3′. Primer specificity was performed as previously described in Ding, Walton, Zhu, and Frisina (2018). Triplet repeated quantitative PCR (qPCR) experiments were executed by creating a master mix using the following primers, SSoFast EvaGreen (Bio‐Rad, Hercules, CA), and the cDNA samples. The samples were then placed in a CFX™ Real Time PCR system to generate a quantitative analysis of the gene expressed in both SVK‐1 cells and SV tissue samples. It should be noted that the relative standard curve method was used when analyzing the data for the following samples.

### 4.5 ELISA assay

Blood samples were extracted from animals after undergoing HRT treatment for 6 months and a recovery/washout period that lasted 1 month. The blood was set to clot in lukewarm water for ~15 min and then centrifuged for 20 min to allow separation. The serum was then collected and stored at −80°C for future use. Serum IGF‐1 concentrations were quantitatively assessed for all of the following samples utilizing manufacture's protocol for an IGF‐1 Quantikine ELISA Kit (R&D Systems, Minneapolis, MD, USA). It should be noted that an intra‐assay precision technique was implemented when the following experiment was performed.

### 4.6 Data statistical analysis

*GraphPad Prism7* was used to perform the statistically analysis for the following experiments. Statistical tests, such as 1‐way ANOVA, 2‐way ANOVA, and Welch's *t* test, were used, depending on the design of the experiment. Bonferroni pairwise comparisons are reported when the main effects of the ANOVA F statistics are statistically significant. Results were identified as statistically significant if *p* < 0.05. A more detailed description about the statistical analysis can be found in the previous publication Williamson et al. (2015).

## CONFLICT OF INTEREST

None declared.

## AUTHORS' CONTRIBUTIONS

T.T.W., B.D., X.Z., and R.D.F. designed the research; T.T.W., B.D., and X.Z. performed the experiments; T.T.W., B.D., R.D.F., and X.Z. analyzed the data; and T.T.W., B.D., and R.D.F. wrote the article.

## DATA AVAILABILITY

The data that support the results for this particular investigation are available upon request from the corresponding author.
