# Supplementary material for: Hormone replacement therapy attenuates hearing loss: Mechanisms involving estrogen and the IGF‐1 pathway
Source: Aging Cell. 2019 Mar 7;18(3):e12939. doi: 10.1111/acel.12939 (PMC6516159; doi:10.1111/acel.12939)
Supplement: Supplementary file 1 [file ACEL-18-e12939-s001.pdf]

Supplementary Material

Table S1. ABR statistics for the HRT study subject groups.

Frequency (kHz)	Baseline vs. 3 Months	Baseline vs. 6 Months	Baseline vs. Recovery
**3**	E: ns<br>P: ns<br>E+P: ns<br>Pb: *<br>Males: ns	E: ns<br>P: ns<br>E+P: ns<br>Pb: ***<br>Males: ns	E: ns<br>P: ns<br>E+P: ns<br>Pb: ***<br>Males: ns
**6**	E: *<br>P: ns<br>E+P:<br>Pb: **<br>Males: *	E: ns<br>P: *<br>E+P: ns<br>Pb: **<br>Males: *	E: ns<br>P: ****<br>E+P: ****<br>Pb: ***<br>Males: *
**12**	E: ns<br>P: ns<br>E+P: ns<br>Pb: ns<br>Males: ns	E: ns<br>P: *<br>E+P: ns<br>Pb: ns<br>Males: ns	E: ns<br>P: **<br>E+P: **<br>Pb: ****<br>Males: **
**16**	E: ns<br>P: ns<br>E+P: ns<br>Pb: ns<br>Males: ns	E: ns<br>P: *<br>E+P: ns<br>Pb: *<br>Males: ns	E: ns<br>P: **<br>E+P: *<br>Pb: ****<br>Males: **
**20**	E: *<br>P: ns<br>E+P: ns<br>Pb: ns<br>Males: ns	E: ns<br>P: **<br>E+P: ns<br>Pb: **<br>Males: ns	E: ns<br>P: ***<br>E+P: *<br>Pb: ****<br>Males: **
**24**	E: ns<br>P: ns<br>E+P:<br>Pb: ns<br>Males: ns	E: ns<br>P: **<br>E+P: ns<br>Pb: ***<br>Males: ns	E: ns<br>P: ****<br>E+P: **<br>Pb: ****<br>Males: ***
**32**	E: ns<br>P: **<br>E+P: ns<br>Pb: ns<br>Males: ns	E: ns<br>P: ***<br>E+P: ns<br>Pb: ***<br>Males: ns	E: ns<br>P: ****<br>E+P: **<br>Pb: ****<br>Males: ***
**36**	E: ns<br>P: **<br>E+P: ns<br>Pb: ns<br>Males: ns	E: ns<br>P: *<br>E+P: ns<br>Pb: **<br>Males: ns	E: ns<br>P: ****<br>E+P: ***<br>Pb: ****<br>Males: *
**48**	E: ns<br>P: ns<br>E+P: **<br>Pb: ns<br>Males: ns	E: ns<br>P: *<br>E+P: ns<br>Pb: *<br>Males: ns	E: ns<br>P: ***<br>E+P: ns<br>Pb: ***<br>Males: **

Table S1. Continued

Frequency (kHz)	Baseline vs. 3 Months	Baseline vs. 6 Months	Baseline vs. Recovery
**WBN**	E: ns<br>P: ns<br>E+P: ns<br>Pb: *<br>Males: ns	E: ns<br>P: ns<br>E+P: ns<br>Pb: **<br>Males: ns	E: ns<br>P: ****<br>E+P: ***<br>Pb: ****<br>Males: **

Shows the statistical differences among the HRT groups for the ABR thresholds at the various checkpoints at which the animals were tested throughout the study. Statistical test: 2 way ANOVA followed by Bonferroni pairwise comparisons corrected for multiple comparisons; *P<0.05, **P<0.01, ***P<0.001, ****P<0.0001.

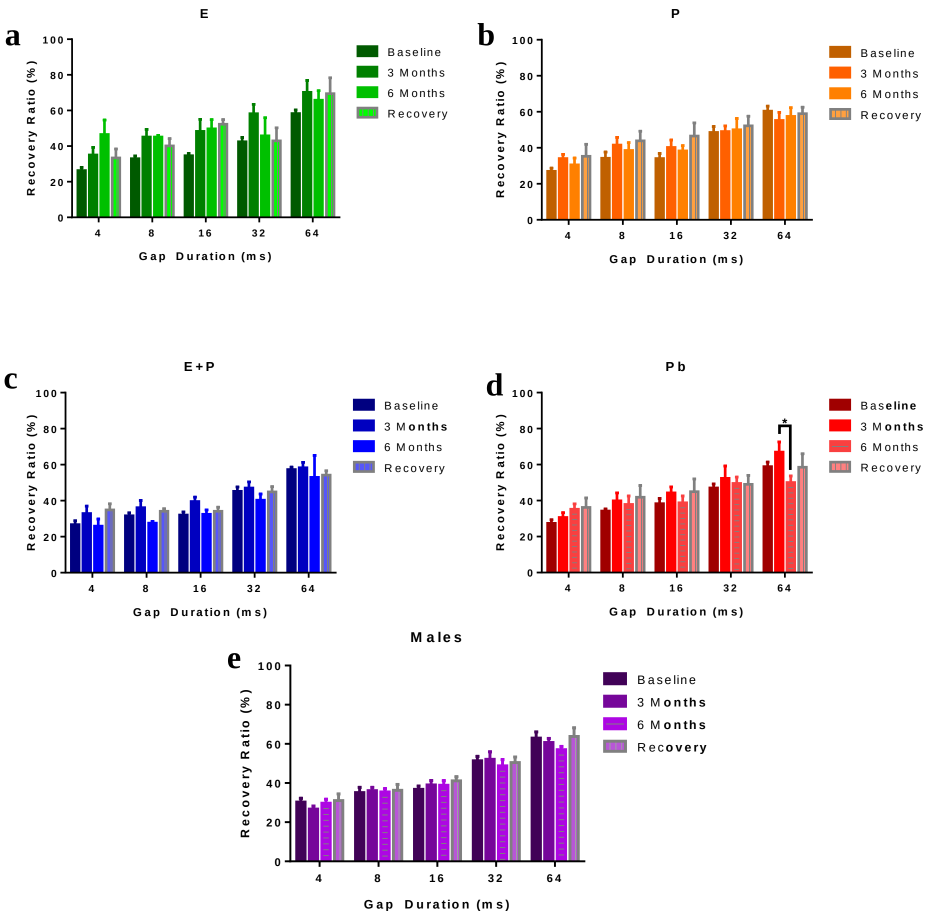

Figure S1. *P1 recovery ratios for NB2 for HRT groups during and after treatment.* Notable differences were not seen for the recovery ratios for almost all of the groups. The (d) Pb animals were the only group to show significant changes between the 3 month and 6 month checkpoint at 64 ms. As for (a) E, (b) P, (c) E+P and (e) the males groups, little to no changes were observed for recovery ratios. Additionally, E treated animals displayed some signs of improvement in recovery ratios during and after HRT. It should be noted that signs of recovery after treatment was discontinued was not observed for any HRT group. Statistical test: 2 way ANOVA followed by Bonferroni; *P<0.05.

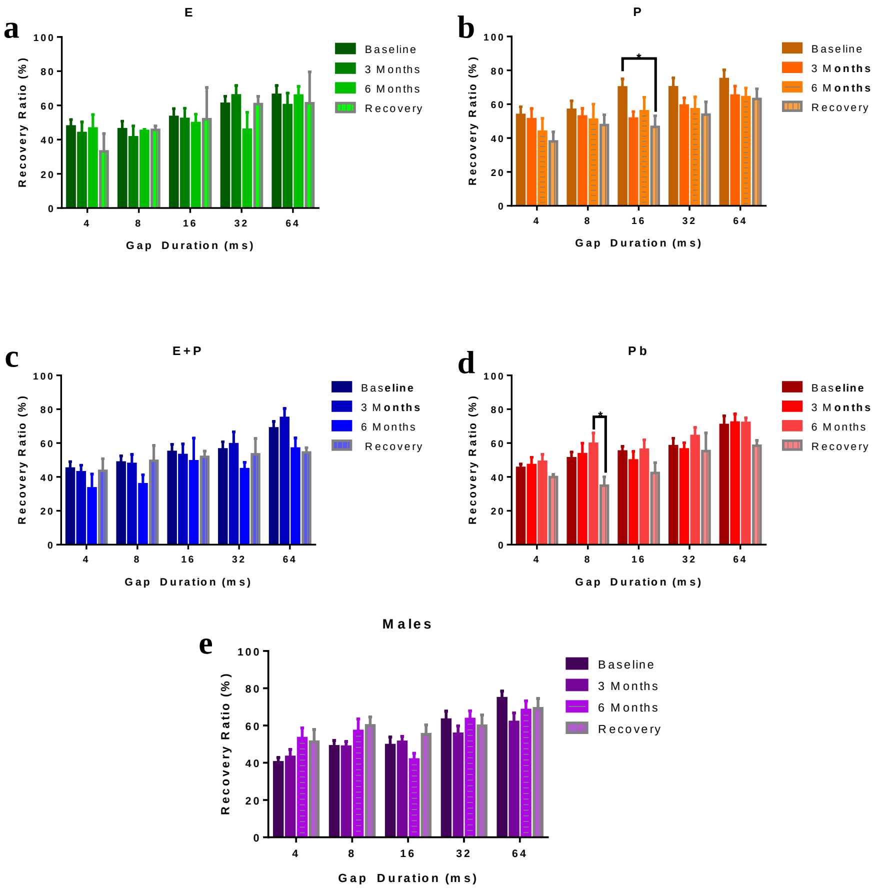

Figure S2. *P4 recovery ratios for NB2 during hormone therapy and for the recovery period.* Significant differences were demonstrated for the (b) P group for baseline vs. recovery at 16 ms and for (d) Pb animals for 6 month vs. recovery at 8 ms. Like the P1 data, there were only slight changes in the (a) E group. Ratio values for the (b) P groups gradually declined with as treatment progressed. Signs of regression mostly began to take place for the (c) E+P group after 6 months of treatment, and declines were seen for (d) Pb animals during the recovery period. (e) The male group had ratios that were relatively the same at larger gap intervals. Auditory recovery was not observed for any of the animals after treatments were stopped. Statistical test: 2 way ANOVA followed by Bonferroni; *P<0.05.

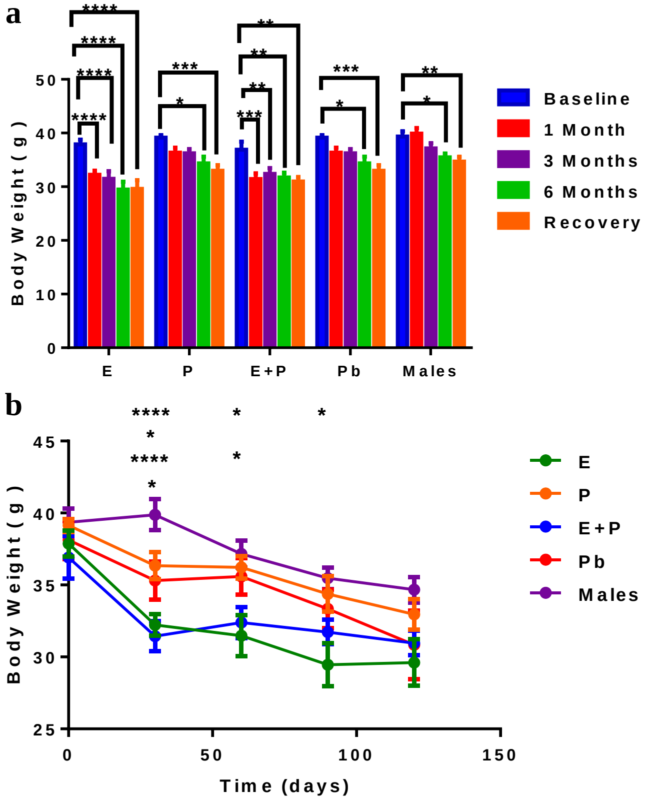

Figure S3. *Animal body weights declined with age throughout the course of the experiment.* (a) P and Pb animals exhibited the smallest declines in body weights during the 6 months of HRT. E and E+P treated animals displayed significant body weight changes 1 month (30 days) into hormone therapy. The body weight for both groups continued to progressively decline from there. (b) The body weight for P animals seemed to be the most consistent throughout the experiment compared to the other groups. This group also had the highest body weight among the female groups for the duration of experiment. Pb animals' body masses showed a parallel trend to the P group; however, weight values dropped more after 6 months (90 days) of treatment. Interestingly, the E group had the lowest body weight, specifically after 1 month (30 days) of HRT, consistent with the notion that body weights are often correlated to improved sensorimotor functioning during aging. Statistical test: 2 way ANOVA followed by Bonferroni; *$P<0.05$, **$P<0.01$, ***$P<0.001$, ****$P<0.0001$.
